# Development of Natural-Drugs-Based Low-Molecular-Weight Supramolecular Gels

**DOI:** 10.3390/gels7030105

**Published:** 2021-08-04

**Authors:** Xiaoyi Feng, Yuning Luo, Fangjie Li, Xueting Jian, Yang Liu

**Affiliations:** Hunan Provincial Key Laboratory of Tumor Microenvironment Responsive Drug Research, Hunan Province Cooperative Innovation Center for Molecular Target New Drug Study, School of Pharmacology, Hengyang Medical School, University of South China, Hengyang 421001, China; fxy5040@163.com (X.F.); luo1748058392@163.com (Y.L.); 18382038762@163.com (F.L.); jxt15872415729@163.com (X.J.)

**Keywords:** natural drug, low molecular weight supramolecular gel, self-assembly, hydrogel, organogel

## Abstract

Natural small molecular drugs with excellent biocompatibility, diverse pharmacological activities, and wide sources play an increasingly important role in the development of new drug and disease treatment. In recent years, the utilization of paclitaxel, camptothecin, rhein, curcumin, and other natural small molecular drugs with unique rigid backbone structures and modifiable multiple sites as building blocks to form gels by self-assembly has attracted widespread attention. The obtained low-molecular-weight supramolecular gel not only retains the general characteristics of the gel but also overcomes the shortcomings of natural drugs, such as poor water solubility and low bioavailability. It has the advantages of high drug loading, low toxicity, and outstanding stimulus responsiveness, which is widely used in biomedical fields. Here, we provided a comprehensive review of natural-drugs-based low-molecular-weight supramolecular gels reported in recent years and summarized their assembly mechanism, gel structure, gel properties, and potential applications. It is expected to provide a reference for further research of natural-drugs-based supramolecular gels.

## 1. Introduction

The gel is a kind of soft material with a three-dimensional network structure formed by crosslinking of colloidal particles, small molecular substances, or polymer chains under certain conditions, which has attracted much attention in the biomedical area, such as drug delivery, tissue engineering, and biosensors [1,2,3]. According to the difference of internal solvents, the gel can be divided into hydrogel, organogel, ionogel, and so on [4,5]. Besides, according to the composition and formation mechanism, we can also divide the gel into macromolecular gel and supramolecular gel [6]. Among them, supramolecular gels are mostly formed from low-molecular-weight molecules, oligomers, or polymers through non-covalent interactions, such as electrostatic interaction, hydrogen bonding, van der Waals force, hydrophobic interaction, host–guest interaction, and so on, or dynamic covalent bonds [7,8,9]. Compared with macromolecular gels, supramolecular gels generally have the advantages of definite structural composition, easy chemical modification, lower critical gelation concentration, and reversible gel formation upon certain external stimulus, which have shown excellent prospects in the biomedical field [10,11,12].

With the further development of research, supramolecular gels formed from natural small molecular drugs have attracted much attention from researchers [13,14]. The natural small molecular drug is a kind of substance derived from biological metabolism, which generally refers to the effective components of traditional Chinese medicine, such as paclitaxel, camptothecin, rhein, curcumin, and so on. Because of its extensive pharmacophore, unique stereochemical structure, multiple sites for modification, and rich sources, it is considered to be one of the great repositories of new drug development [15,16,17]. However, most natural drugs also have some disadvantages, including poor water solubility and low bioavailability. To improve their therapeutic effect, researchers have developed various carriers for the delivery of natural drugs, such as nanoparticles, micelles, liposomes, and polymer gels [18,19,20]. Among them, polymer gels have attracted increasing research interest in natural drug delivery because of their outstanding biocompatibility, viscoelasticity, controlled and sustained drug release performance, and certain targeting properties [21,22,23]. Nonetheless, it also has some limitations, such as low drug loading, incomplete degradation, and so on. To solve these problems, some studies directly used natural drug molecules as building blocks to self-assemble into low-molecular-weight supramolecular gels, which are further used to deliver drugs to lesion sites and exert their efficacy. In 1977, Acree et al. [24] inadvertently found that cholesterol can be self-assembled in isopropanol to form a transparent gel in the process of determining the solubility of cholesterol in organic solvents, which may be the first found low-molecular-weight supramolecular gel based on natural drug. In 2009, Gao et al. [25] took the paclitaxel (PTX) as an example and firstly established a general preparation method of nanofiber supramolecular hydrogel drug delivery system based on hydrophobic drug molecules. In 2011, Bag et al. [26] discovered that betulinic acid could self-assemble to form gels in some organic solutions, which revealed the face of the first supramolecular gel based on a triterpene drug.

**Figure 1:**
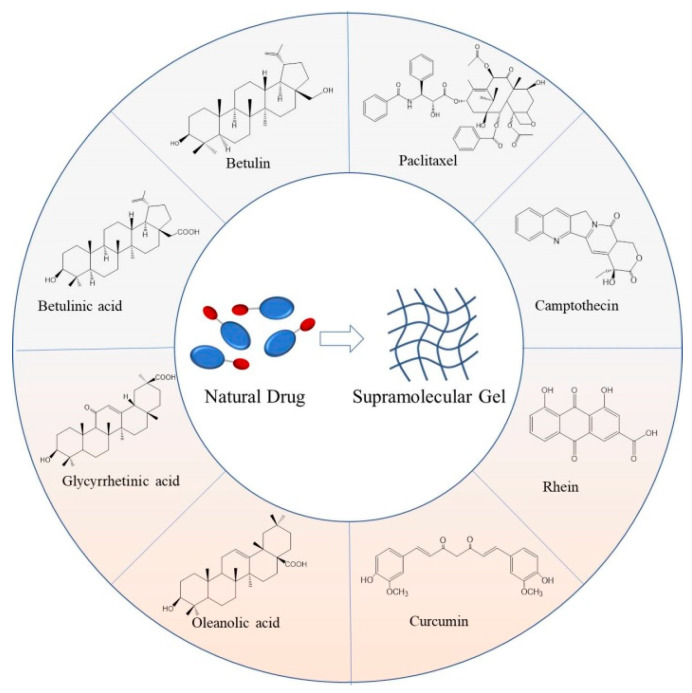
The structure schematic of reported representative natural drugs and the corresponding low-molecular-weight supramolecular gels formed from them.

With the deepening development of related research, as a new type of self-assembly system, low-molecular-weight supramolecular gel based on natural drugs has become a hot spot in the current interdisciplinary research, attracting extensive attention from researchers. The formed supramolecular gels are both a delivery vehicle and a drug, which not only improve the solubility and stability of small molecular natural drugs, but also ensure the formed gels have excellent performance, such as lower toxicity, higher drug loading, and reversible stimulus-response. Moreover, they also have a good application prospect in other fields, such as water treatment, biosensing, and nanocatalysis. However, as far as we know, there are no reviews of natural-drugs-based low-molecular-weight supramolecular gels in the literature. So, here we comprehensively summarized a representative number of the most extensively studied natural drugs that could form low-molecular-weight supramolecular gels, including paclitaxel, camptothecin, rhein, curcumin, and so on (Figure 1). The structure, preparation, and application of reported low-molecular-weight supramolecular gels based on these natural drugs were expounded. The development trend and future challenges of this kind of gel were also prospected.

## 2. Paclitaxel-Based Low-Molecular-Weight Supramolecular Gels

Paclitaxel (PTX) or taxol is a kind of tricyclic diterpenoid compound with high antitumor activity isolated from Taxus brevifolia. It is widely used in the treatment of breast cancer, ovarian cancer, and lung cancer, which is one of the most attractive anti-tumor star drugs in the clinical treatment [27]. Paclitaxel has a unique anti-tumor mechanism. By binding to tubulin and preventing its depolymerization, paclitaxel can effectively inhibit the mitosis of tumor cells, prevent the progress of the cell cycle, and then promote the apoptosis of tumor cells [28]. However, it is suffered from low water solubility, which seriously restricts the anti-tumor effect in vivo. Although a variety of paclitaxel-based preparations were put on the market or in the stage of clinical research, the construction of novel paclitaxel-based drug delivery systems is still one of the hotspots at home and abroad.

**Figure 2:**
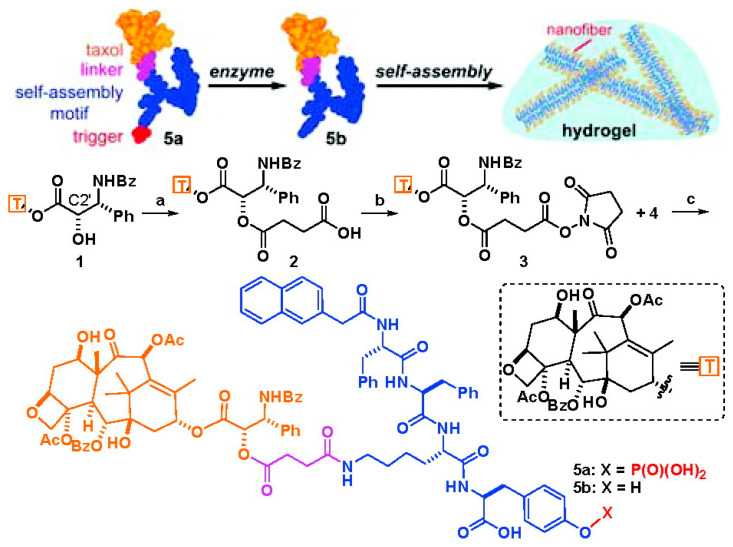
The schematic diagram of the formation of the first paclitaxel-derivative-based supramolecular nanofiber hydrogel obtained by the use of short peptides, and the preparation process of this paclitaxel derivative. Reprinted with permission from Copyright (2009) American Chemical Society.

Short peptides have a large number of amino and carboxyl groups, which present good biocompatibility and rich structural diversity, and are often used to modify and regulate the hydrophilicity and hydrophobicity of natural small molecular drugs. Besides, through reasonable design, short peptides are easily assembled into hydrogels using non-covalent forces such as hydrogen bonding and π–π stacking, which have aroused extension attention in the construction of supramolecular gels. As mentioned above, the first paclitaxel-based low-molecular-weight nanofiber supramolecular hydrogel delivery system was obtained by the use of short peptides [25]. As shown in Figure 2, the author first introduced a carboxyl group into paclitaxel by esterification of C2′ hydroxyl group with succinic anhydride, and then a paclitaxel derivative with better solubility and water stability was obtained by amide condensation with a phosphatase substrate NapFFKYp short peptide. Under the stimulation of alkaline phosphorylase, the paclitaxel derivative can be dephosphorylated and further rapidly self-assembled to form a nanofiber hydrogel under non-covalent interaction. The obtained hydrogel could slowly release paclitaxel derivative with the same physiological activity as PTX and was able to inhibit the proliferation of HeLa cells. This study provided a simple and versatile formation method of hydrogel for enzyme-mediated self-assembly of hydrophobic drug molecules. Another study also found that the low concentration of paclitaxel released from this type of paclitaxel hydrogel not only enhanced neurite elongation as free paclitaxel did, but also promoted the branches of axons by inhibiting the depolymerization of tubulin which was not achieved by using free paclitaxel [29]. Furthermore, by simultaneously coupling doxorubicin [30], folic acid [31], dexamethasone [32], or embedding vorinostat [33] and tetrandrine [34] on short peptides, the researchers can further regulate the properties of paclitaxel-based supramolecular gels and improve the therapeutic effect. At the same time, through the accurate molecular design of the charge of the short peptide and the type of amino acid, the stability or dissociation of the formed paclitaxel-based hydrogel can be adjusted, and then the release of paclitaxel can be controlled, which can improve the anti-tumor effect and reduce the systemic toxicity [35]. 

However, since the formation of the above-mentioned paclitaxel-based hydrogel requires the participation of short peptides, the design and preparation of this gel precursor will take a longer time and higher cost. Besides, the gelation concentration is difficult to adjust and the drug loading is also limited. Therefore, the direct usage of paclitaxel or its simple derivatives to construct supramolecular hydrogels has attracted much attention. Wang et al. [36] first used succinic anhydride to couple paclitaxel with hydrophilic oxidized glutathione (GSH) through an ester bond to improve water solubility. When the conjugate is put into the PBS buffer (pH = 7.4) due to the slow hydrolysis of the ester bond, a paclitaxel-based supramolecular nanofiber hydrogel will be obtained within 6 h. In vivo animal experiments showed that the gel can effectively inhibit tumor growth and metastasis. The GSH used in this method can be replaced by other hydrophilic molecules, so it is expected to be used in the clinical treatment of tumors. For example, Luo et al. [37] replaced GSH with RGD peptide and obtained a paclitaxel-based supramolecular hydrogel which could effectively release paclitaxel for more than one week. 

While Song et al. [38] found that PTX-succinic acid derivatives synthesized by the esterification reaction of PTX with succinic anhydride could also be self-assembled into transparent hydrogels by the ionization of carboxyl groups and hydrolysis of ester bonds in the PBS buffer. This hydrogel exhibited a microscopically filamentous nanostructure and had good injectability and self-healing properties. In vitro drug release experiments showed that the drug release behavior could be regulated by changing the initial concentration of the gel, and the drug release time could be up to 15 days. The CCK-8 cytotoxicity test also showed that the gel could effectively inhibit the proliferation of HepG2 liver cancer cells and MCF-7 breast cancer cells, and its concentration was positively correlated with the cell toxicity. The supramolecular hydrogel has good thixotropy and is easy to be obtained, so it might be a promising vehicle for local anti-cancer therapy. Yang et al. [39] also found that the mechanical strength of this gel could be increased by adding a certain amount of hyaluronic acid. As the content of hyaluronic acid increases, the anti-tumor effect of the paclitaxel hydrogel could be significantly improved. 

Zhang et al. [40] adopted a simpler method to obtain an injectable supramolecular hydrogel by self-assembly under the action of π–π stacking and hydrogen bonding, in which paclitaxel was directly used as raw material. Paclitaxel was first dissolved in polyethylene glycol (PEG) with a molecular weight of 200 or 400. Then, a small amount of deionized water, PBS buffer, or normal saline was slowly added to it, and the formation of paclitaxel-based hydrogel could be observed after a certain period. This formation method of paclitaxel-based gels is simple and efficient, with easily adjustable gelation concentration and high drug loading capacity. Moreover, it is convenient to introduce other drugs into this hydrogel to play the role of synergistic treatment. This gel has good injectable properties and can maintain stability for more than 40 days at the injection site of mice. Compared with the paclitaxel injection and normal saline, it can significantly inhibit tumor growth and show less systemic toxicity. 

As a traditional anticancer drug, paclitaxel is often used in combination with other anticancer drugs to treat tumors in clinical practice. To overcome the drug resistance of tumor cells to paclitaxel and the cytotoxicity caused by the degradation and metabolism of drug carriers in vivo, Ren et al. [41] used paclitaxel and hydrophilic tyroservatide (YSV) to prepare amphiphilic dual-drug molecules by the coupling of succinic anhydride. When the ester bond slowly underwent self-hydrolysis in the aqueous solution, the released free paclitaxel could be assembled with the unhydrolyzed dual-drug molecules to form a nanofiber supramolecular hydrogel. The maximum gelation concentration of this paclitaxel gel was more than 1 wt%. The paclitaxel could be released slowly in vitro with no obvious burst release phenomenon, and the sustained release time was longer than 7 days. Cell experiments and animal experiments also showed that, due to the synergistic effect of tyroservatide, the cumulative absorption of paclitaxel in tumor cells increased, and the dual-drug hydrogel had a stronger anti-tumor effect and lower side effect.

## 3. Camptothecin-Based Low-Molecular-Weight Supramolecular Gels

Camptothecin (CPT) is an alkaloid active substance extracted from the traditional Chinese medicine Camptotheca acuminata with a quinoline ring as the basic core. Because of its inhibitory activity of DNA topoisomerase I, CPT is a commonly used broad-spectrum anticancer drug in the clinic [42]. Similar to paclitaxel, CPT has low water solubility and stability, making it difficult to use directly and safely in the body. To solve this problem, researchers often modify the active site on the structure of camptothecin (such as the hydroxyl group on the E-ring) to a certain extent and construct various new drug delivery systems to improve its solubility and anti-tumor activity [43].

**Figure 3:**
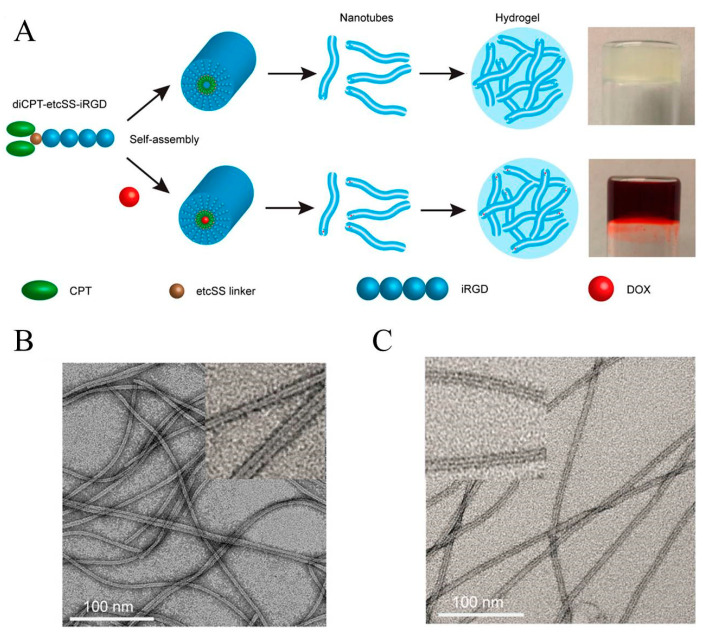
Schematic illustration of the camptothecin-based amphiphile tubustecan (TT6) hydrogel as chemotherapeutic carrier: (**A**) the chemical design, self-assembly, and drug loading of the hydrogel; (**B**) Representative TEM images of the hydrogel and (**C**) DOX-loaded hydrogel. Reproduced with permission from Copyright (2020) American Chemical Society.

The Cui group coupled two camptothecin molecules to the same short peptide and constructed a camptothecin analogue tubustecan (TT) [44]. Due to hydrogen bonding and other effects, tubustecan can cyclize and self-assemble to form tubular supramolecular nanostructures in an aqueous solution, which improves the water solubility of camptothecin and can be further used to load other hydrophobic drugs or molecules. In a representative study, they used a circular cell penetrating peptide iRGD to prepare a camptothecin-based tubustecan (TT6) through the coupling of biodegradable ethyl disulfonyl carbonate (etcSS linker) [45]. As shown in Figure 3, TT6 could first self-assemble to form a hollow tubular structure in an aqueous solution, and further assemble into a nanofiber hydrogel after the adding of PBS or DMEM medium. It was found that, when doxorubicin was embedded into the hydrogel, the resulting hydrogel could slowly release doxorubicin and TT6 with zero-order release characteristics. Besides, under the action of reductive GSH, TT6 could further quickly release CPT. In vivo experiments also showed that the gel can be formed quickly at the injection site, maintaining a sustained release of the drug for at least 45 days, and can improve the penetration and anti-tumor ability of the drug. When curcumin was introduced into this hydrogel, it was also found that it could inhibit the growth of primary breast cancer and prevent it from metastasizing to the lungs. The group also used TT as an immune enhancer for tumor immunotherapy based on the immune checkpoint blocker (ICBs) [46]. They used an amphipathic prodrug diCPT-PLGLAG-iRGD to prepare supramolecular gel. Because of the presence of PLGLAG polypeptide fragment, this hydrogel can be broken under the action of the over-expressed MMP-2 metalloproteinase in the tumor microenvironment, thus accelerating the release of CPT and the blended aPD1 antibody to induce the body to produce long-lasting anti-tumor immunity and thereby inhibit tumor recurrence and metastasis.

Besides, the utilization of short peptides to prepare camptothecin-short peptide derivatives, which are then assembled to form camptothecin-based supramolecular hydrogels, has also attracted considerable interest from researchers. Song et al. [47] reported the CPT-short peptide derivative hydrogels with self-assembled nanotube structures for the first time. In this report, FFYGE-ss-EEE-short peptides with broken disulfide bonds were synthesized by solid phase peptide synthesis and then coupled with glycine-linked CPT to obtain CPT-G-Succ-FFYGE-ss-EEE derivatives. After the addition of GSH for about 20 min, due to the rupture of the disulfide bond, the resulting CPT-G-Succ-FFYGE-SH can be assembled to form a hydrogel with a unique nanotube structure. This hydrogel can be stable at room temperature for more than 3 months, and can slowly release CPT through ester bond hydrolysis. Compared with free CPT, the gel had a lower IC_50_ value for HepG2 hepatoma cells and the same IC_50_ value on normal mouse 3T3 fibroblasts. The study is expected to be applied to the clinical treatment of cancer. Schiapparelli et al. also used a bioreducible disulfanyl butyrate (buSS) linker to couple camptothecin and the hydrophilic short peptide to obtain a amphiphilic gel prodrug CPT-HKD, which can self-assemble to form filaments in an aqueous solution [48]. Under the action of PBS or salts, this filament can further form supramolecular hydrogels by self-assembly. In vitro experiments showed that the supramolecular hydrogel can slowly release CPT-HKD at a linear release rate, and can also release CPT quickly under the action of GSH. By constructing an orthotopic GBM resection, a recurrence mice model, and injecting CPT-HKD solution, it was found that it could immediately form a gel in situ and that the gel significantly delayed tumor recurrence.

10-hydroxycamptothecin (HCPT) has a stronger anticancer effect than CPT, but also has low water solubility. Inspired by the aforementioned hydrogels of CPT-short peptide derivatives, Wu et al. [49] also prepared an HCPT-SA-FFEssEE gel precursor to improve the water solubility of HCPT by the use of HCPT and a disulfide bond-containing polypeptide FFEssEE as raw materials and succinic anhydride (SA) as a coupling agent. When GSH is added to its aqueous solution, the resulting HCPT-SA-FFE gel factor rooted in the rupture of the disulfide bond can be self-assembled to form a translucent hydrogel by π–π stack effect. The hydrogel can continuously release HCPT through the hydrolysis of ester bonds to ensure a higher drug concentration. Cell experiments showed that its anti-tumor ability was significantly better than free HPCT. The gel has slender fiber network structures, good recovery performance, and shear thinning ability, which is expected to be used in injectable tumor local drug delivery systems. 

## 4. Rhein-Based Low-Molecular-Weight Supramolecular Gels

Rhein (Rh), a kind of anthraquinone compound extracted from the traditional Chinese medicine rhubarb, has a wide range of pharmacological effects. It can inhibit the proliferation and induce apoptosis of malignant cells such as ovarian cancer, breast cancer, and liver cancer [50]. It also has anti-inflammatory, bacteriostatic, antiviral, and other functions. The structural modification of rhein to improve its efficacy, solubility, and other physical and chemical properties has become the focus of current research.

**Figure 4:**
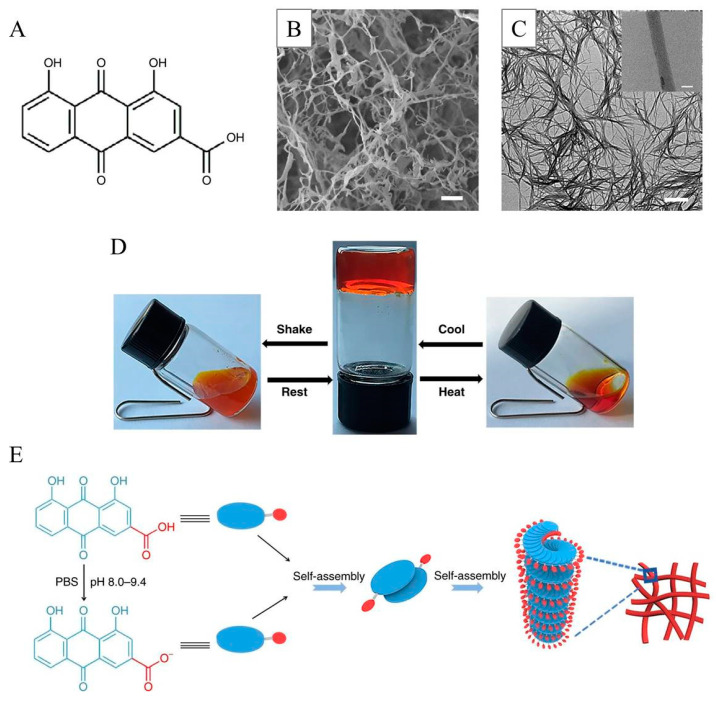
Schematic illustration of the rhein-based hydrogel: (**A**) Chemical structure of rhein; (**B**) SEM image of the rhein hydrogel; (**C**) TEM image of the rhein hydrogel; (**D**) Reversible sol–gel transitions of rhein-based hydrogel triggered by shear stress and temperature; (**E**) Self-assembly mechanism of the rhein-based hydrogel. Reproduced with permission from Copyright (2019) Nature Publishing Group.

Zheng et al. [51] found that rhein could be self-assembled directly into an orange-red nanofiber supramolecular hydrogel with a three-dimensional network structure after a simple heating–cooling process (Figure 4). The hydrogel has certain stimulus responsiveness, and can undergo a reversible sol-gel transition when the temperature and pH value are changed. The gel can release rhein slowly, and it is easier to enter cells and bind to the TLR4 receptor on the cell surface, thereby significantly inhibiting dephosphorylating IκBα and the p65 nuclear metastasis in the NFκB signaling pathway of BV2 microglia induced by lipopolysaccharide, and alleviating neuroinflammatory response for a long time. The authors believed that the π–π stacking between anthraquinones and the hydrogen bonding and the electrostatic interaction between molecules play an important role in the stable formation of this hydrogel. Feng et al. [52] also conducted a detailed study on the formation of this rhein-based hydrogel through molecular simulation and considered that the ionization of the carboxyl group of rhein at different pH conditions plays a crucial role in the formation of this gel.

To further improve the stability and anti-inflammatory effect of rhein in the gel, Zhao et al. introduced a second crosslinking network to the rhein nanofiber supramolecular gel [53]. Firstly, the hyaluronic acid derivative modified by ferrocene and derivative modified by β-cyclodextrin were prepared and then mixed with rhein solution, respectively. After further mixing of the two mixtures for 30 min, a hyaluronic acid/rhein composite gel will form by the host–guest interaction of ferrocene and β-cyclodextrin. Due to the cracking of ferrocene under oxidation conditions, the obtained hydrogels can respond to the oxidative microenvironment of inflammatory wounds and regulate the release of rhein accordingly. The supramolecular gel can successfully promote the transition of chronic wounds from the inflammation stage to the normal wound healing stage, and promote the repair of chronic wounds.

Besides, rhein also has a good anti-tumor ability. So Xu et al. [54] established a supramolecular hydrogel containing double anticancer drugs of rhein and cisplatin (Pt) to improve the anticancer effect. Firstly, rhein was modified with short peptides to obtain an amphiphilic rhein–peptide coupling compound Rh-GFFYERGD, which can be self-assembled to form fibers. Because the two carboxyl groups of aspartic acid (D) on the short peptide had strong chelating action on platinum ions, the cisplatin that was added later could further promote the assembly of Rh-GFFYERGD fibers to form three-dimensional supramolecular hydrogels. It was found that, after treating A549 cells with this rhein-based gel, rhein could be better absorbed by the cells and accumulated in the nucleus. The gel can slowly release rhein and cisplatin, thus showing better synergistic anti-tumor ability.

## 5. Curcumin-Based Low-Molecular-Weight Supramolecular Gels

Curcumin (Cur), a hydrophobic polyphenol compound extracted from the rhizome of turmeric, has various pharmacological effects such as antioxidant, anti-inflammatory, hypoglycemic, antihyperlipidemic, neuroprotection, and so on. Clinical experiments also demonstrated that curcumin could inhibit the generation, transformation, proliferation, and metastasis of tumor cells, while has little toxicity to normal cells [55]. Because of a strong conjugation structure formed by two benzene rings and heptadienedione units, curcumin also has the disadvantage of high molecular rigidity, small water solubility, photothermal sensitivity, and low water stability, which greatly limit its applications in clinical practice.

**Figure 5:**
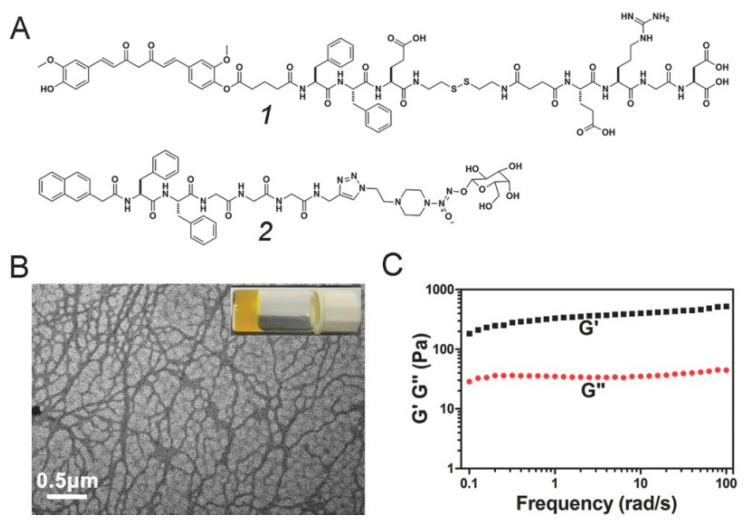
Schematic illustration of the curcumin-based mixed component supramolecular hydrogel: (**A**) Chemical structure of compounds **1** and **2**; (**B**) TEM image and optical image of the mixed component hydrogel; (**C**) Dynamic frequency sweep of the mixed component hydrogel (strain = 1%). Reproduced with permission from Copyright (2017) Wiley-VCH GmbH.

To overcome these shortcomings, Yang et al. [56] reported the first curcumin derivative-based supramolecular hydrogel in 2014. Firstly, the short peptide FFE-ss-ERGD containing disulfide bond was coupled with Cur by using glutaric anhydride as a coupling agent to obtain Cur-FFE-ss-ERGD precursor compound (compound **1** in Figure 5), which could be converted into Cur-FFE-SH gelator under the reduction in GSH. The formed Cur-FFE-SH gelators could be transformed into a yellowish hydrogel in situ under physiological conditions. The obtained supramolecular gel has an intertwined filamentous nanofiber structure, which can release Cur slowly due to the hydrolysis of the ester bond in PBS, and is expected to be used in the local treatment of cancer. Moreover, a kind of gel precursor Nap-FFGGG-NO which could produce NO (compound **2** in Figure 5) was mixed with the above Cur-FFE-ss-ERGD precursor compound 1. Under the action of GSH, a mixed supramolecular gel capable of releasing Cur and NO together would be obtained for the treatment of myocardial infarction. Through intramyocardial injection, the combination of curcumin and NO was found to significantly reduce collagen deposition, improve cardiac function, inhibit apoptosis and hypertrophy, and promote poor myocardial remodeling, showing a promise in the treatment of cardiovascular diseases [57]. To improve the targeting ability of the curcumin-based hydrogel, the group also introduced glycyrrhetinic acid (GA) into short peptide through amidation reaction and further obtained GA-GFFYK(Cur)E-ss-ERGD (GA-Cur) gel precursor [58]. Because GA can specifically bind to the over-expressed GA receptor protein kinase C on the membrane of liver cancer cells, the curcumin gel precursor has active targeting properties and could enter the liver cancer cells through endocytosis. Under the action of the high content of GSH inside the cell, the disulfide bond of the precursor will be broken, and then a hydrogel will be formed in situ by self-assembly. Cur can be slowly released by hydrolysis of ester bonds. The results suggested that the targeted gels with higher cellular uptake and anti-tumor efficacy are promising approaches for the treatment of liver cancer.

Xu et al. [59] also used a curcumin-short peptide derivative-based supramolecular hydrogel as a high-performance radiosensitizer for the first time. The precursor compound of Cur-FFE-CS-EE was synthesized, and then a nanofiber hydrogel was obtained by self-assembly under the action of GSH. Studies found that the gel can inhibit the activation of nuclear factor κB (NF-κB) and the production of cytokine IL-6/TNFα induced by radiation cancer cells, thereby increasing the sensitivity of colon cancer cells to ionizing radiation and inhibiting their proliferation. In vivo experiments showed that the combination of the gel and physical radiation could significantly reduce the tumor volume of tumor-bearing mice. 

Besides, Granata et al. [60] constructed an injectable supramolecular gel for the sustained release of curcumin using the interaction between curcumin and calixarene derivatives. Firstly, a cationic amphiphilic derivative of choline-calixarene was prepared, which could self-assemble into micelles under physiological conditions. Then curcumin powders were added to the solution and dissolved by ultrasound. With the slow dissolution of curcumin, a reddish hydrogel will eventually be produced. The study showed that curcumin acts as a crosslinking agent in the formation of hydrogels by electrostatic interaction and hydrogen bonding. The obtained gel has good self-healing properties, allowing it to self-adapt to any shape of space inside the injection site. It also can effectively stabilize curcumin, improve the solubility of curcumin, avoid chemical and photothermal degradation, and slowly release the calixarene micelles loaded with curcumin. Furthermore, the research group investigated the application of this curcumin-based nano-hydrogel in the treatment of psoriasis by constructing a psoriasis-like IMQ-induced skin inflammation model [61]. The authors found that the curcumin-based gel could restore the normal distribution of TJs protein ZO1 and occludin, reduce the expression of TNF-α, and inducible nitric oxide synthase (iNOS), indicating that the curcumin-based supramolecular gel can improve the therapeutic effect of psoriasis and have a good application prospect.

## 6. Oleanolic Acid Based Low-Molecular-Weight Supramolecular Gels

Oleanolic acid (OA) is a well-known pentacyclic triterpene with an oleanol skeleton, which exists in many plants in free form or combined into glycosides. At present, it is mainly isolated and extracted from the fruits of Gentianaceae plant Gentiana or Ligustrum lucidum. OA has a curative effect on liver protection and has been used in the clinic for many years. Furthermore, OA has a variety of pharmacological activities, such as hypoglycemic, antioxidant, anticancer, antiviral, anti-platelet aggregation, and so on [62].

Currently reported oleanolic-acid-based supramolecular gels are mostly formed in organic solvents by modifying the carboxyl or hydroxyl groups of OA to promote non-covalent interactions between molecules. For example, Hu et al. [63] prepared the first oleanolic-acid-based supramolecular gel by self-assembling 2,3-dioxime oleanolic acid in aromatic solvents or chlorinated alkanes through a heating–cooling process. The results showed that the intermolecular hydrogen bonding between the carboxyl group and the oxime group was the main driving force for the formation of this organogel. Lu et al. [64] obtained an oleanolic acid conjugate by coupling adenine to the hydroxyl group at the 3-position of the OA skeleton. This conjugate could not form a gel in tetrahydrofuran (THF), but when water was gradually added to it, the solution changed from clarification to opaque gel and then to flocculent precipitation. The authors believed the reason is that the addition of water destroyed the hydrogen bond between the N-H of the adenine atom in the conjugated molecule, enhanced its solubility in THF, and made the solution clear from turbidity. After that, the molecules were rearranged under the intermolecular hydrogen bond and the hydrophobic action of the triterpene skeleton, and then self-assembled to form the gel. Adding the complementary base uracil to the gel system can break the hydrogen bond of adenine, which would cause the dissociation of the gel and realize the responsive recognition of special molecules. The author also obtained an oleanolic acid-short peptide conjugate by modified the A-ring hydroxyl group of OA with a short peptide, which can also be self-assembled to form a gel through hydrogen bonds and van der Waals forces in solvents such as benzene, toluene, or xylene [65]. After drying, the gel can be used to adsorb dyes such as rhodamine 6 G and acridine yellow from water, so it is expected to be used in wastewater purification treatment. 

**Figure 6:**
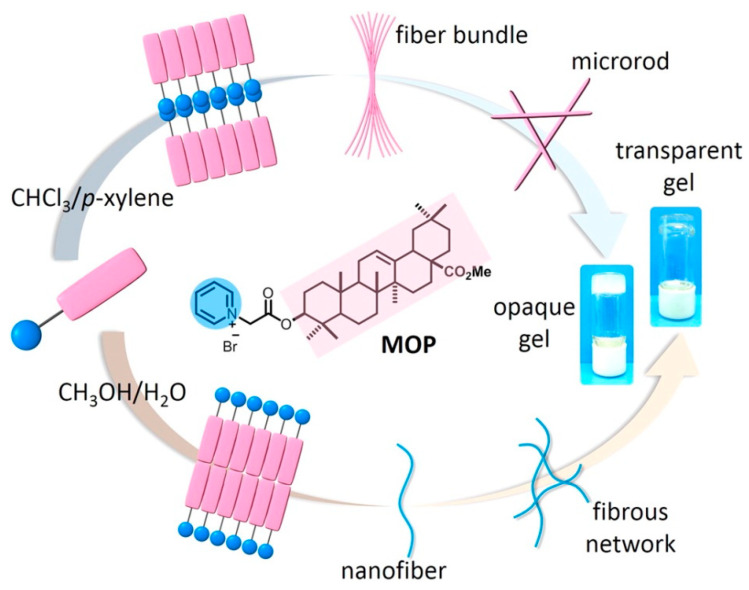
Schematic illustration of the solvent-directed assembly of oleanolic-acid-based amphiphilic derivative MOP in chloroform/p-xylene and methanol/water systems, respectively. Reprinted with permission from Copyright (2016) American Chemical Society.

Gao et al. [66] further studied in detail the effect of solvent polarity on the assembly of oleanolic acid derivatives (Figure 6). The author first modified the A ring of ethyl oleanolic acid with pyridinium to obtain the amphiphilic derivative MOP and then studied its self-assembly using chloroform/p-xylene or methanol/water mixed solvents. The results showed that, due to the influence of solvation, van der Waals force, and other non-covalent interaction, with the addition of p-xylene in a chloroform/p-xylene solvent system, MOP first assembled into spherical nanoparticles, gradually transformed into microrods, and finally formed an opaque gel. While, in methanol/water solvent, with the addition of water, MOP changed from spherical nanoparticle to nanofiber and finally formed a transparent gel. This research provided a basis for the assembly of natural drugs. Vega-Granados et al. [67] also modified the aromatic ring on the carboxyl group of the OA skeleton and then dissolved the resulting derivative in hot organic solvents. Similar to the previous method, the authors found that the derivative can also be quickly assembled into an organogel when cooled in the mixed solvents of DMSO/water or DMF/water. The authors also found that the DMSO or DMF solution of this derivative could spontaneously acquire moisture in the air, and then slowly self-assembled into a gel with a more uniform structure and greater mechanical strength.

However, the above methods all require structural modification of oleanolic acid, and the formation of gel requires a delicate balance between various forces, such as hydrophilicity and hydrophobicity, intermolecular interaction, and so on. Therefore, researchers are increasingly interested in the direct self-assembly of natural small molecules into gels without any moieties or structural modifications. OA has a rigid multi-ring skeleton structure, and the carboxyl and hydroxyl groups on the skeleton have certain polarity, so it is possible to directly assemble into a gel. Bag et al. [68] reported for the first time that OA molecules could be directly self-assembled into vesicles by heating and cooling in 11 aromatic solvents, straight-chain alcohols of C3–C5 and C7, chloroform, carbon tetrachloride, DMSO/water mixture, and other solvents, and then stacked into thermally reversible gels. The main driving force of assembly is the hydrogen bond formed by carboxyl and hydroxyl groups in OA molecules. The obtained hydrogel can not only be used to encapsulate the rhodamine B fluorescent molecule but also as a drug carrier to deliver the anticancer drug doxorubicin.

Recently, Fan et al. [69] designed and prepared the first OA-based low-molecular-weight supramolecular hydrogel in water by heat induction. They first prepared a simple salt of OA and choline by neutralization reaction. After heating, this salt solution would turn into the hydrogel. The higher the concentration of this salt and temperature were, the easier it was for the salt solution to form a hydrogel. The authors thought the reason was that heating would reduce the salt–water hydrogen bonding interactions, and thus, decrease the solubility of the salt, resulting in the formation of network gels under the drive of the hydrophobic interactions and van der Waals. This supramolecular hydrogel was stable and injectable, and has potential to be the drug delivery vehicles for the sustained release of hydrophilic drugs.

## 7. Glycyrrhetinic Acid Based Low-Molecular-Weight Supramolecular Gels

As a valuable traditional Chinese medicine, the main active ingredient of Glycyrrhiza uralensis Fisch is glycyrrhetinic acid (GA) and its diglucuronide glycyrrhizic acid [70]. Glycyrrhetinic acid, similar to oleanolic acid, also belongs to oleanane-type pentacyclic triterpene, which has a variety of biological activities, such as anticancer, anti-inflammatory, antiviral, antibacterial, and liver protection, and its structure is easy to modify. Therefore, there are an increasing number of related studies in recent years.

Glycyrrhetinic acid has a special five-ring three terpene structure, in which the A ring and E ring have hydroxyl and carboxyl substituents, respectively, and the C ring has an alkene structure, so it may be directly self-assembled into an organogel. In 2012, Bag et al. [71] reported the first glycyrrhetinic-acid-based supramolecular organogel. The author found that, after heating and dissolving glycyrrhetinic acid in some specific organic solvents, a translucent gel could be obtained after the cooling process. These solvents include 13 kinds of common aromatic solvents, ethylene glycol, glycerol, aliphatic chlorinated hydrocarbons, and mixed solvents of DMSO/water and DMF/water. The hydrogen bonding between hydroxyl and carboxyl groups in the structure of glycyrrhetinic acid plays an important role in the formation of the gel. The obtained organogel has a flower-like spherical or sphere-like microstructure and can be used as a template for the formation of CdS nanoparticles. The Ju group also modified the hydroxyl group of A-ring of methyl glycyrrhetinate with the pyridine group and obtained a new amphiphilic MGP molecule, which could be self-assembled into spiral nanofibers by π–π stacking interaction, van der Waals interaction, and hydrophilic interaction in chloroform/aromatic solvents [72]. This nanofiber can further assemble into a transparent chiral organogel through mutual entanglement. This study provided a simple and effective method for the construction of supramolecular chiral assemblies. The group also prepared an organogel through the charge transfer effect of pyrene modified glycyrrhetinic acid and 2,4,7-trinitrofluorenonefluorine in mixed solvents of DMSO/water or DMF/water [73].

However, the formation of the above-mentioned glycyrrhetinic-acid-based supramolecular gel requires the use of organic solvents, which greatly limits the application of glycyrrhetinic acid in the biomedical field. To solve this problem, the Ju group reported the first glycyrrhetinic-acid-based supramolecular hydrogel in 2013 [74]. The authors found that an opaque and thermally reversible hydrogel could be formed by slightly heating and dissolving a certain amount of sodium glycyrhizinate in an alkaline aqueous solution with pH > 9 and then cooling. Further research has proved that the electrostatic interaction and dipole–dipole interaction between the sodium carboxylate were the main driving forces for the formation of hydrogels, and the special skeleton structure of glycyrrhetinic acid also played an essential role in it. Because of the negative charge, the hydrogel can be used to adsorb and remove positively charged dyes such as pyridine yellow and rhodamine 6 G in the solution. Moreover, they constructed the first glycyrrhetinic-acid-based supramolecular hydrogel which could form in PBS physiological buffer by the self-assembly of pyridine modified glycyrrhetinic acid amphiphilic molecule (GP) [75]. The GP was obtained by the modification of the carboxyl group of E-ring of glycyrrhetinic acid with pyridine by the bridging effect of PEG. By heating and dissolving GP in PBS and cooling it, a medium strength physical hydrogel could be obtained. The π–π stacking interaction between pyridine rings, the electrostatic interaction between phosphate ions and pyridine, van der Waals interaction, and hydrophobic interaction played a synergistic role in the formation of this hydrogel. The hydrogel had no obvious toxicity to 3T3-L1 cells and could be used to load and release the anti-tumor drug DOX in situ under physiological conditions. This group also used uracil modified glycyrrhetinic acid methyl ester to construct a kind of hydrogel that could respond to F^−^ and Hg^2+^ in an aqueous solution by hydrogen bonding and π–π stacking interaction [76]. Using the host–guest interaction between glycyrrhetinic acid and β-cyclodextrin, this group also constructed a self-healing glycyrrhetinic-acid-based hydrogel [77]. 

Besides, supramolecular gels based on glycyrrhizic acid have also attracted the attention of many researchers. Mezzenga et al. [78] directly constructed a glycyrrhizic-acid-based hydrogel through a simple method. A certain amount of glycyrrhizic acid was dissolved in hot water and placed at room temperature, and then a transparent nanofiber hydrogel can be obtained through self-assembly. The microstructure study showed that the hydrophobic triterpene skeleton of glycyrrhizic acid was horizontally arranged in the internal in a head-to-head manner, while the hydrophilic disaccharide unit was exposed to the outside to form right-handed spiral fibers. A hybrid hydrogel was also constructed by introducing graphene oxide into it. The sugar units in the gel can be used to reduce the chloroauric acid in situ to produce gold nanoparticles and to catalyze the reduction process of nitrophenol to aminophenol. Due to the interaction with the catalytic substrate, the addition of graphene can promote the enrichment of gold nanoparticles to the surrounding substrate and improve the catalytic rate. This study provided a new idea for the use of natural supramolecular gels in the construction of multi-system catalytic composites. 

**Figure 7:**
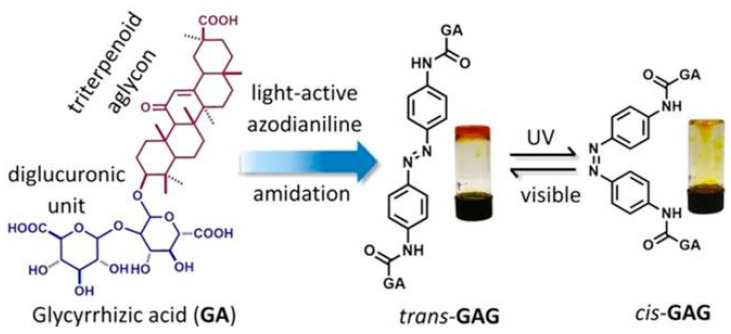
Schematic representation of the gelation of trans-GAG and its reversible trans–cis isomerization. Reprinted with permission from Copyright (2018) Wiley-VCH GmbH.

Fang et al. [79] connected two molecules of glycyrrhizic acid to both sides of trans-azobenzene through amide bonds to form a trans-GAG gel precursor (Figure 7). It could be self-assembled into a gel in both DMSO/H_2_O and MeOH/H_2_O mixed solvents. Due to the presence of trans-azobenzene, the gel would undergo isomerization under UV and visible light irradiation, resulting in reversible dissociation of the gel. The gel has outstanding biocompatibility characteristics, which can maintain cell growth for a long time. It was also self-healing and could easily be transferred into a syringe and extruded to create desired patterns on the substrate by 3D printing. Wan et al. [80] also used the nanofibers assembled by glycyrrhizic acid as stabilizers to establish an oil-in-water emulsion based on vegetable oil at high temperature by one-step emulsification. After cooling, a kind of emulsion gel, based on glycyrrhizic acid fiber, will form. This gel has a thermosensitive behavior and is expected to be used in a stable drug delivery system. 

## 8. Betulinic Acid/Betulin-Based Low-Molecular-Weight Supramolecular Gels

Betulinic acid and betulin are lupane pentacyclic triterpenoids extracted from the bark of jujube and birch trees, which have anti-tumor, anti-cancer, anti-diabetes, and other pharmacological activities. The difference in chemical structure between betulinic acid and betulin only lies in the substituent at C-28, which is also the reason why betulinic acid has a higher biological activity than betulin [81].

Due to its unique structural characteristics, betulinic acid can be directly assembled to form a gel. It was found that betulinic acid can be assembled to form nanofiber organogels in 19 common aromatic solvents, alcohol solvents, and so on [26]. The low water solubility of betulinic acid hinders the formation of betulinic-acid-based supramolecular hydrogels. To overcome this problem, Bag et al. [82] used the more hydrophilic sodium and potassium salts of betulinic acid as the gel building blocks for research. The results showed that two kinds of salts could form opaque organogels in DMSO/water, ethanol/water, and other mixed solvents. At the same time, two kinds of salts could also be directly assembled in water to form nanofiber hydrogels. The obtained hydrogel had good stability and could be sealed for several months at room temperature. The authors believed that the main forces for the formation of the gel were the electrostatic interaction and the dipole–dipole interaction between molecules. This charged hydrogel can adsorb rhodamine B, fluorescein, neutral red, and other colored dyes, and is expected to be applied to wastewater treatment. Moreover, because jujube bark contains phenolic compounds that could reduce Au (III) to Au (0), the author also used jujube bark extract and Au (III) colloid mixed with two kinds of salts, respectively, and in-situ synthesized gold nanoparticles doped by gel hybrid materials.

Betulin can also be directly assembled to form a gel without any structural modification. Bag et al. [83] found that betulin could be assembled into opaque gels in o-xylene, m-xylene, p-xylene, DMSO/water, or other solvents by hydrogen bonding. Microstructural studies showed that all the gel self-assemblies had symmetrical flower-like structures with a diameter of nanometers. The gel had a porous microstructure and could absorb and remove toxic dyes such as methylene blue and crystal violet. Besides, the gel can also be used as a biocompatible carrier to deliver doxorubicin and other drugs, which is expected to be used in the drug delivery system.

## 9. Other Natural-Drugs-Based Low-Molecular-Weight Supramolecular Gels

Ursolic acid (UA) is a pentacyclic triterpenoid compound of ursolic type. Similar to oleanolic acid, it also has a variety of pharmacological activities such as anti-tumor, anti-oxidation, anti-virus, and so on [84]. Lu et al. [85] found for the first time that ursolic acid can be self-assembled to form an organogel in organic solvents, such as bromobenzene, without any modification. The intermolecular hydrogen bonds and triterpene skeletons play an important role in the formation of gels. Furthermore, the author introduced the aromatic ring into the molecular structure of ursolic acid through the connection of the amide bond. Using the π–π stacking effect of the aromatic ring and the hydrogen bonding effect of the amide bond, ursolic acid can also self-assemble into a transparent nanofiber organogel in other aromatic solvents, such as chlorobenzene, which provided a new idea for the design of supramolecular gels [86].

Puerarin is a kind of isoflavone derivative isolated from traditional Chinese medicine Pueraria lobata. It has antipyretic, sedative, and crown-dilating effects, and is mainly used in the treatment of coronary heart disease, angina pectoris, and hypertension [87]. Cai et al. [88] directly used the self-assembly of puerarin to form a supramolecular gel with excellent oxidation resistance and acid resistance through a heating–cooling operation. The gel could overcome the injury of exogenous reactive oxygen species and improve the survival rate of H_2_O_2_-treated cells through the down-regulated activity of superoxide dismutase and the content of malondialdehyde in bone marrow mesenchymal stem cells. The dissociation rate of the puerarin-based gel in simulated intestinal juice was also significantly faster than that in simulated gastric juice, indicating that the puerarin-based gel is expected to be used in oral preparations. However, some studies also found that the obtained gel also has some limitations such as poor mechanical strength and thermal stability [89]. Given this, Li et al. [90] used N-(9-fluorenylmethoxycarbonyl)-L-phenylalanine (Fmoc-Phe-OH) to blend with puerarin to form a double interpenetrating network structure of Fmoc-Phe-OH nanofibers and puerarin, resulting in an enhanced puerarin-based hydrogel. The mechanical strength and thermal stability of this gel have been greatly improved, and it still has pH responsiveness. At the same time, because it contains Fmoc in the structure, it has antibacterial properties. The gel can load and release the antibacterial model drug berberine hydrochloride, thus playing a synergistic antibacterial effect.

In addition, due to the essential role of carbohydrate compounds in life activities, the low-molecular-weight supramolecular gels based on carbohydrates have also received great attention [91,92]. For example, Bielejewski et al. [93,94,95] used methyl-4,6-O-(p-nitrobenzylidene)-α-D-glucopyranoside as the gelators and prepared a series of supramolecular gels. They found that this unique saccharide gelators could form hydrogel in water, or organogels in some usual organic solvents by a heating–cooling process. Especially, this gelators could also form a recoverable ionogel with potential applications in electrochemical devices in the ionic liquid tetramethylammonium bromide by self-assembly of hydrogen bonding. Oosumi et al. [96] also designed a type of bola-amphiphilic glycolipid-type supramolecular hydrogelators via a one-pot reaction between various 4-aminophenyl monosaccharide derivatives and 2,3-dichloromaleimide derivatives bearing a carboxy group. These monosaccharides included α-D-galactose, β-D-galactose, α-D-glucose, β-D-glucose, and α-D-mannose. These prepared hydrogelators will form hydrogels by self-assembly during a heating–cooling cycle, which could also exhibit color changes along with the gel–sol transition. However, it should be mentioned that, since many small molecular carbohydrates, mainly monosaccharides and disaccharides, are not usually used directly as drugs, there are only a few reports on the application of low-molecular-weight supramolecular gels based on carbohydrates in the medical field, such as wound healing [97] and cell culture [98].

Besides the natural drugs mentioned above, other supramolecular gels based on coumarin [99], anthracene [100], arjunolic acid [101], poricoic acid A [102], and soybean sterol [103] also showed great research and application prospects, which attracted the attention of a large number of researchers.

## 10. Conclusions and Perspectives

Natural small molecular drugs have attracted much attention in the field of new drug development and pharmaceutics because of their diverse pharmacological activities, wide sources, and good biocompatibility. Besides, many of them have a unique stereoscopic rigid structure, rich in hydroxyl and carboxyl groups and other easily modified sites, so there are strong non-covalent interactions among molecules, including hydrogen bonding, π–π stacking, dipole–dipole interaction, hydrophobic interaction, and van der Waals force. Under the influence of these non-covalent interactions, these natural small molecular drugs are easy to aggregate and arrange, so they are expected to form low-molecular-weight supramolecular hydrogels, organogels, or ionogels through the assembly. The obtained natural-drugs-based supramolecular gels combine the advantages of gel and medicine, which are both a delivery carrier and a drug. These gel materials can not only overcome the shortcomings of natural small molecular drugs such as low solubility, low bioavailability, and poor stability, but also have lower toxicity, higher drug loading, and stimulation response release, which have attracted wide attention in the field of drug controlled release, wound repair, and disease treatment. Besides, they have also been applied in wastewater treatment, chemical sensing, and nanocatalysis, and so on. Here, we reviewed the latest research progress of reported gels formed from natural drugs, including paclitaxel, camptothecin, rhein, curcumin, etc., and summarized their formation mechanism, gel structure, gel properties, and potential applications, which will provide references for the research of natural-drugs-based supramolecular gels.

At present, researchers have developed and prepared various kinds of natural-drugs-based low-molecular-weight supramolecular gels with excellent properties through ingenious design. These obtained supramolecular gels generally have a fibrous microstructure. According to whether the crosslinking reaction in gels occurs directly on the structural fragment of natural small molecular drugs, we can roughly classify these supramolecular gels into two groups. In the first type of supramolecular gels, the crosslinking reaction did not take place directly on the structural fragment of natural drug molecules. Researchers usually first use some hydrophilic bioactive groups to modify the natural drugs to improve the water solubility of the molecules, and then the gel that is usually a hydrogel will be formed by the interaction of the bioactive groups. The commonly used bioactive groups are originated from short peptides, which have easily modified structures and adjustable properties. Through the use of short peptides with special properties, such as enzyme responsive or redox responsive, rational design of the charges and amino acids of short peptides, or even coupling with other drugs or bioactive molecules, the performance of these supramolecular gels can be adjusted, thereby enhancing the treatment efficacy of natural drugs and reducing the side effects. This method of forming supramolecular gel is widely applicable, and the gel properties are easy to adjust, but the cost is high, and the drug loading is small. 

The second kind of natural-drugs-based supramolecular gels is mainly directly crosslinked by the non-covalent interactions between the structural fragments of natural drugs through controlling the nature of natural drugs, including concentration, solubility, molecular structure, and so on. To obtain these types of natural-drugs-based supramolecular hydrogels, researchers usually modify the natural drug molecule with a hydrophilic group by a cleavable chemical bond (such as ester bond, disulfide bond, etc.) to make the drug dissolve in water firstly. Furthermore, with the slow breakage and departure of the hydrophilic group, the solubility of the natural drug gradually decreases. Due to the strong non-covalent interactions between drug molecules, the molecules do not precipitate or crystallize but form a hydrogel through self-assembly. While, to obtain an organogel or ionogel based on a natural drug, the natural drug molecule is usually dissolved in a hot organic solvent or ionic liquid first, and then, a gel may form by crosslinking during the slow cooling of this solution. This gel-forming method is relatively simple, and the content of the drug is very high. However, this method needs to go through a lot of tedious repeated experiments, which has a great contingency.

However, the development of the natural-drugs-based low-molecular-weight supramolecular gels is still in its infancy, and there are still many problems to be further studied and solved. (1) The amount of natural small molecular drugs that can be assembled to form supramolecular gels is still not enough. It is still difficult for many natural small molecular drugs with unique chemical structures to form a gel or to carry out relevant research. Therefore, we should strive to discover more natural-drugs-based supramolecular gels, thereby expanding the scope of this kind of supramolecular gels. (2) The formation of supramolecular gels based on natural drugs has a great contingency. Therefore, we should thoroughly study the molecular structure of natural drugs and the basic laws and properties of the gel formed from them, and develop more general gel preparation method. (3) Many of the natural-drugs-based supramolecular gels have been reported to be organogels or ionogels, which are difficult to give full play to the bioactivity advantages of natural drugs. Therefore, we should do more researches on the preparation and application of supramolecular hydrogels based on natural drugs. Besides, we should also further strengthen the research to ensure the obtained natural-drugs-based supramolecular gels have better biocompatibility and stability, and fully expand their application range from drug-controlled release to other biomedical fields.

## Data Availability

The study did not report any data.
